# Reliability of Serum Metabolite Concentrations over a 4-Month Period Using a Targeted Metabolomic Approach

**DOI:** 10.1371/journal.pone.0021103

**Published:** 2011-06-15

**Authors:** Anna Floegel, Dagmar Drogan, Rui Wang-Sattler, Cornelia Prehn, Thomas Illig, Jerzy Adamski, Hans-Georg Joost, Heiner Boeing, Tobias Pischon

**Affiliations:** 1 Department of Epidemiology, German Institute of Human Nutrition Potsdam-Rehbruecke, Nuthetal, Germany; 2 Institute of Epidemiology, Helmholtz Zentrum München, German Research Center for Environmental Health, Neuherberg, Germany; 3 Institute of Experimental Genetics, Genome Analysis Center, Helmholtz Zentrum München, German Research Center for Environmental Health, Neuherberg, Germany; 4 Department of Pharmacology, German Institute of Human Nutrition Potsdam-Rehbruecke, Nuthetal, Germany; 5 Molecular Epidemiology Group, Max Delbrück Center for Molecular Medicine (MDC) Berlin-Buch, Germany; Governmental Technical Research Centre of Finland, Finland

## Abstract

Metabolomics is a promising tool for discovery of novel biomarkers of chronic disease risk in prospective epidemiologic studies. We investigated the between- and within-person variation of the concentrations of 163 serum metabolites over a period of 4 months to evaluate the metabolite reliability expressed by the intraclass-correlation coefficient (ICC: the ratio of between-person variance and total variance). The analyses were performed with the BIOCRATES Absolute*IDQ*™ targeted metabolomics technology, including acylcarnitines, amino acids, glycerophospholipids, sphingolipids and hexose in 100 healthy individuals from the European Prospective Investigation into Cancer and Nutrition (EPIC)-Potsdam study who had provided two fasting blood samples 4 months apart. Overall, serum reliability of metabolites over a 4-month period was good. The median ICC of the 163 metabolites was 0.57. The highest ICC was observed for hydroxysphingomyelin C14:1 (ICC = 0.85) and the lowest was found for acylcarnitine C3:1 (ICC = 0). Reliability was high for hexose (ICC = 0.76), sphingolipids (median ICC = 0.66; range: 0.24–0.85), amino acids (median ICC = 0.58; range: 0.41–0.72) and glycerophospholipids (median ICC = 0.58; range: 0.03–0.81). Among acylcarnitines, reliability of short and medium chain saturated compounds was good to excellent (ICC range: 0.50–0.81). Serum reliability was lower for most hydroxyacylcarnitines and monounsaturated acylcarnitines (ICC range: 0.11–0.45 and 0.00–0.63, respectively). For most of the metabolites a single measurement may be sufficient for risk assessment in epidemiologic studies with healthy subjects.

## Introduction

The “omic” sciences – including genomics, proteomics and metabolomics, among others - are promising novel approaches that may be useful in prospective epidemiologic studies to screen various targets at once with the aim to identify candidate biomarkers for the estimation of the risk of chronic diseases, such as cardiovascular disease or diabetes. Metabolomics systematically identifies and quantifies low-molecular weight compounds that are intermediates or endpoints of metabolism. Because metabolites may change rapidly in response to physiologic perturbations, they may represent more proximal reporters of intermediary or disease phenotypes than e.g. proteins [1], [2], [3]. Expanding the view from the more static genomic and proteomic fields to metabolomics may, therefore, reveal more insight into a system that is more sensitive to external stimuli. Thus, metabolomics is a promising technique for candidate biomarker discovery to assess chronic disease risk in large-scale epidemiologic studies [4]. To gain a reliable risk estimate with a single blood measurement, as is usually obtained in large epidemiologic studies, the within-subject variance over time should be small compared with the between-subject variance since poor reliability generally tends to bias relative risks in epidemiologic studies between biomarkers and disease risk towards the null [5]. Thus, the high sensitivity of the metabolome to internal or external stimuli (such as age, hormonal status, diet and lifestyle) may potentially limit their use for risk assessment in large-scale epidemiologic studies that are based on single blood measurements [6], but little is known on their within- and between-person variance.

To address this issue, the present study aimed to evaluate the reliability, expressed by the intraclass-correlation coefficient (ICC) [5], as the ratio of between-person variance and total variance, of 163 targeted metabolites in fasting serum samples over a 4-month period within a sub-sample of the European Prospective Investigation into Cancer and Nutrition (EPIC)-Potsdam cohort. This metabolomic approach has already been successfully applied in the human KORA cohort study [7], [8], and covers acylcarnitines, amino acids, glycerophospholipids, sphingolipids and hexoses, constituting a biologically relevant panel of 163 metabolites.

## Results

The total sample consisted of 100 healthy participants who were evenly distributed according to gender with a mean age of 56.1 years (**Table 1**). Men were older, had a higher BMI, waist-circumference and waist-to-hip ratio than women.

**Table 1:** Characteristics of the EPIC-Potsdam Sub-Sample.

	All (n = 100)		Men (n = 50)		Women (n = 50)	
	Mean	(SD)	Mean	(SD)	Mean	(SD)
Age (years)	56.1	(4.05)	57.9	(3.13)	54.4	(4.15)
BMI (kg/m^2^)	26.8	(4.04)	28.0	(3.78)	25.6	(3.96)
Waist (cm)	94.6	(13.5)	103	(10.9)	86.1	(10.1)
Waist-to-hip ratio	0.89	(0.10)	0.96	(0.06)	0.81	(0.06)

Mean serum metabolite concentrations ranged from 0.01 µmol/L for a number of acylcarnitines to 5207 µmol/L for hexose. On average, the serum reliability of 163 metabolites was good with a median ICC of 0.57. The highest reliability was observed for hydroxysphingomyeline SM(OH)C14:1 (ICC = 0.85) and the lowest was found for acylcarnitine C3:1 (ICC = 0). The ICCs for the individual metabolites according to their subclasses are presented in **Tables 2** to [Table pone-0021103-t003] [Table pone-0021103-t004] **5** (**Table 2**: acylcarnitines; **Table 3**: amino acids, lysoglycerophospholipids, sphingolipids and hexose; **Table 4**: diacyl-glycerophospholipids, and **Table 5**: acyl-alkyl-glycerophospholipids). Excellent reliability (ICC≥0.75) was found for hexose; hydroxysphingomyelins SM(OH)C14:1, SM(OH)C22:2 and SM(OH)C16:1; sphingomyelins SMC18:1 and SMC16:1; phosphatidylcholines PCaeC44:6, PCaeC42:5, PCaeC36:2 and PCaeC42:4; and Butyryl-L-carnitine (C4). Among the metabolites that were poorly reliable (ICC<0.4), there were 16 out of 41 acylcarnitines, besides 14 out of 92 glycerophospholipids and sphingomyelines SMC22:3, SMC20:2 and SMC26:0.

**Table 2:** Geometric Means, Variances and Intraclass-Correlation Coefficients (ICCs) of Serum Concentrations of Acylcarnitines Measured 4 Months Apart Among 100 Healthy Subjects from the EPIC-Potsdam Cohort.

	1st Measurement		2nd Measurement			CV (%)			
Metabolite [µmol/L]^a^ ^b^	Mean	95% CI	Mean	95% CI	*P-value* c	Within-person	Between-person	ICC^d^^,^ e ^,^ ,f	95% CI
**C0**	35.8	(34.1–37.6)	37.0	(35.3–38.8)	0.09	13.4	19.7	0.69	(0.57–0.78)
**C10**	0.35	(0.32–0.38)	0.37	(0.33–0.41)	0.20	27.0	38.5	0.67	(0.55–0.77)
**C10:1** g	0.21	(0.20–0.22)	0.23	(0.21–0.24)	0.04	20.4	23.2	0.57	(0.42–0.68)
**C10:2**	0.04	(0.04–0.04)	0.04	(0.04–0.04)	0.44	21.0	19.2	0.46	(0.29–0.60)
**C12** g	0.13	(0.12–0.14)	0.14	(0.13–0.15)	0.09	23.1	23.0	0.50	(0.34–0.63)
**C12-DC** g	0.03	(0.03–0.04)	0.04	(0.03–0.04)	0.16	16.8	7.2	0.15	(0.00–0.34)
**C12:1** g	0.03	(0.03–0.04)	0.04	(0.03–0.04)	0.25	19.6	17.9	0.45	(0.28–0.60)
**C14** g	0.10	(0.09–0.10)	0.09	(0.09–0.10)	0.28	14.7	11.5	0.38	(0.20–0.53)
**C14:1**	0.26	(0.25–0.27)	0.27	(0.26–0.28)	0.05	15.5	16.2	0.52	(0.36–0.65)
**C14:1-OH** g	0.02	(0.02–0.02)	0.02	(0.02–0.02)	0.08	25.4	19.7	0.37	(0.19–0.53)
**C14:2**	0.04	(0.04–0.04)	0.04	(0.04–0.04)	0.39	30.7	28.4	0.46	(0.29–0.60)
**C14:2-OH**	0.01	(0.01–0.01)	0.01	(0.01–0.01)	0.17	37.8	20.5	0.23	(0.03–0.40)
**C16**	0.14	(0.13–0.14)	0.14	(0.14–0.15)	0.08	18.7	18.3	0.49	(0.33–0.63)
**C16-OH** g	0.01	(0.01–0.01)	0.01	(0.00–0.01)	0.20	44.8	15.4	0.11	(0.00–0.30)
**C16:1** g	0.05	(0.05–0.06)	0.05	(0.05–0.06)	0.99	17.6	17.1	0.49	(0.32–0.62)
**C16:1-OH** g	0.01	(0.01–0.01)	0.01	(0.01–0.01)	0.72	32.9	13.8	0.15	(0.00–0.34)
**C16:2**	0.01	(0.01–0.01)	0.01	(0.01–0.01	0.26	40.9	23.2	0.24	(0.05–0.42)
**C16:2-OH** g	0.01	(0.01–0.01)	0.01	(0.01–0.01)	0.02	31.6	12.4	0.13	(0.00–0.32)
**C18**	0.05	(0.05–0.06)	0.06	(0.05–0.06)	0.10	28.1	22.9	0.40	(0.22–0.55)
**C18:1**	0.18	(0.17–0.19)	0.19	(0.18–0.21)	0.00	19.2	18.7	0.49	(0.32–0.62)
**C18:1-OH** g	0.01	(0.01–0.01)	0.01	(0.01–0.01)	0.87	43.4	22.1	0.21	(0.01–0.39)
**C18:2**	0.06	(0.06–0.07)	0.07	(0.07–0.07)	0.00	21.9	23.9	0.54	(0.39–0.67)
**C2**	6.97	(6.55–7.41)	7.18	(6.74–7.65)	0.29	20.1	24.1	0.59	(0.45–0.70)
**C3**	0.39	(0.36–0.41)	0.39	(0.36–0.41)	0.97	18.7	27.9	0.69	(0.57–0.78)
**C3-DC/C4-OH** g	0.04	(0.04–0.04)	0.04	(0.04–0.05)	0.18	17.0	0.23	0.23	(0.04–0.41)
**C3-DC-M/C5-OH**	0.02	(0.02–0.02)	0.02	(0.02–0.02)	0.58	22.6	0.45	0.45	(0.28–0.59)
**C3-OH** g	0.01	(0.01–0.01)	0.01	(0.01–0.01)	0.08	36.7	15.6	0.15	(0.00–0.34)
**C3:1** g	0.01	(0.01–0.01)	0.01	(0.01–0.01)	0.27	57.6	0.00	0.00	
**C4**	0.23	(0.21–0.25)	0.23	(0.21–0.25)	0.81	18.8	38.4	0.81	(0.73–0.87)
**C4:1** g	0.01	(0.01–0.01)	0.01	(0.01–0.01)	0.60	35.1	11.3	0.09	(0.00–0.28)
**C4:1-DC/C6**	0.09	(0.08–0.09)	0.09	(0.08–0.10)	0.13	22.0	30.6	0.66	(0.53–0.76)
**C5**	0.13	(0.12–0.14)	0.14	(0.13–0.15)	<.001	21.2	28.1	0.64	(0.50–0.74
**C5-DC/C6-OH**	0.02	(0.02–0.02)	0.02	(0.02–0.02)	0.80	25.4	0.41	0.41	(0.24–0.56)
**C5-M-DC** g	0.03	(0.02–0.03)	0.03	(0.03–0.03)	0.57	28.7	32.0	0.56	(0.40–0.68)
**C5:1** g	0.04	(0.03–0.04)	0.04	(0.03–0.04)	0.75	26.1	9.4	0.11	(0.00–0.30)
**C5:1-DC** g	0.01	(0.01–0.02)	0.01	(0.01–0.02)	0.68	31.8	11.3	0.11	(0.00–0.30)
**C6:1** g	0.01	(0.01–0.01)	0.01	(0.01–0.01)	0.57	30.1	9.8	0.10	(0.00–0.29)
**C7-DC**	0.04	(0.04–0.04)	0.04	(0.04–0.05)	0.26	26.1	21.7	0.41	(0.23–0.56)
**C8** g	0.26	(0.24–0.28)	0.28	(0.26–0.30)	0.02	20.1	33.5	0.73	(0.63–0.81)
**C8:1**	0.10	(0.10–0.11)	0.12	(0.11–0.13)	0.00	27.9	36.4	0.63	(0.50–0.73)
**C9**	0.04	(0.04–0.05)	0.04	(0.04–0.05)	0.46	26.1	30.8	0.58	(0.44–0.70)

a Abbreviations are as follows: Cx:y (x = number of carbons in the fatty acid side chain, y  =  number of double bonds in the fatty acid side chain), decarboxyl (DC), hydroxyl (OH). For detailed nomenclature see Table S1.

b n = 100.

c A paired t-test based on log-transformed values was calculated to compare geometric means of metabolite concentrations over-time.

d Based on log transformed values.

e ICC calculated as the ratio of between-person variance and total variance.

f For negative values ICC was calculated based on positive variance estimators [33].

g Metabolite concentration was below the assay's limit of detection.

**Table 3 pone-0021103-t003:** Geometric Means, Variances and Intraclass-Correlation Coefficients (ICCs) of Serum Concentrations of Amino Acids, Lysoglycerophospholipids, Sphingolipids and Hexose Measured 4 Months Apart Among 100 Healthy Subjects from the EPIC-Potsdam Cohort.

	1st Measurement		2nd Measurement			CV (%)			
Metabolite [µmol/L]^a^ ^b^	Mean	95% CI	Mean	95% CI	*P-value* c	Within-person	Between-person	ICC^d^^,^ e ^,^ f	95% CI
**Amino Acids**									
** Arg**	135	(130–140)	136	(132–141)	0.49	11.4	13.7	0.59	(0.45–0.70)
** Gln**	726	(701–751)	741	(713–770)	0.22	11.7	14.3	0.60	(0.46–0.71)
** Gly**	313	(298–329)	329	(313–345)	0.01	14.1	20.4	0.68	(0.55–0.77)
** His**	93.7	(90.7–96.9)	96.4	(93.0–100)	0.07	11.0	13.5	0.60	(0.46–0.71)
** Met**	36.5	(35.1–38.0)	37.8	(36.2–39.4)	0.12	15.8	13.2	0.41	(0.24–0.56)
** Orn**	112	(107–118)	121	(116–127)	0.00	15.8	18.4	0.58	(0.43–0.69)
** Phe**	67.1	(64.6–69.7)	70.6	(68.1–73.3)	0.01	13.9	13.1	0.47	(0.30–0.61)
** Pro**	196	(185–208)	206	(194–219)	0.02	15.9	25.6	0.72	(0.62–0.80)
** Ser**	140	(134–146)	147	(141–154)	0.01	13.4	16.7	0.61	(0.47–0.72)
** Thr**	104	(98.7–109)	108	(103–113)	0.08	17.4	15.5	0.44	(0.27–0.59)
** Trp**	83.8	(81.2–86.5)	84.9	(82.2–87.8)	0.44	12.1	10.9	0.45	(0.28–0.59)
** Tyr**	80.1	(76.6–83.8)	84.2	(80.2–88.4)	0.03	15.9	17.4	0.54	(0.39–0.67)
** Val**	338	(324–353)	351	(333–369)	0.09	15.9	17.8	0.56	(0.41–0.68)
** xLeu**	261	(250–274)	276	(263–289)	0.01	15.7	18.1	0.57	(0.42–0.69)
**Lysoglycerophospholipids**									
** lysoPC a C14:0**	3.28	(3.11–3.46)	3.36	(3.13–3.62)	0.47	24.3	21.6	0.44	(0.27–0.59)
** lysoPC a C16:0**	157	(151–163)	167	(160–175)	0.01	15.6	15.1	0.49	(0.32–0.62)
** lysoPC a C16:1**	4.51	(4.24–4.79)	4.69	(4.38–5.02)	0.20	21.7	24.8	0.57	(0.42–0.69)
** lysoPC a C17:0**	2.54	(2.39–2.69)	2.65	(2.49–2.82)	0.09	18.5	24.5	0.64	(0.50–0.74)
** lysoPC a C18:0**	49.8	(47.6–52.1)	52.6	(50.2–55.2)	0.02	16.7	16.5	0.50	(0.33–0.63)
** lysoPC a C18:1**	30.5	(28.9–32.1)	31.8	(30.0–33.6)	0.12	18.7	20.3	0.54	(0.39–0.66)
** lysoPC a C18:2**	36.33	(34.2–38.6)	37.52	(35.0–40.2)	0.29	21.2	25.1	0.58	(0.44–0.70)
** lysoPC a C20:3**	3.29	(3.08–3.50)	3.37	(3.18–3.58)	0.47	24.9	18.7	0.36	(0.18–0.52)
** lysoPC a C20:4**	8.50	(8.00-9.03)	9.11	(8.58–9.67)	0.01	19.8	23.2	0.58	(0.43–0.69)
** lysoPC a C24:0** g	0.37	(0.34–0.39)	0.35	(0.33–0.38)	0.32	25.2	19.3	0.37	(0.19–0.53)
** lysoPC a C26:0** g	0.51	(0.47–0.56)	0.51	(0.47–0.55)	0.91	31.0	32.0	0.52	(0.36–0.65)
** lysoPC a C26:1** g	3.09	(3.03–3.15)	3.05	(2.99–3.10)	0.28	9.1	3.7	0.14	(0.00–0.33)
** lysoPC a C28:0** g	0.48	(0.44–0.51)	0.47	(0.44–0.50)	0.62	24.7	24.1	0.49	(0.32–0.62)
** lysoPC a C28:1**	0.76	(0.71–0.81)	0.73	(0.68–0.78)	0.25	20.6	25.4	0.60	(0.46–0.71)
** lysoPC a C6:0** g	0.02	(0.02–0.02)	0.02	(0.02–0.02)	0.81	48.2	15.1	0.11	(0.00–0.32)
**Sphingolipids**									
** SM (OH) C14:1**	7.08	(6.66–7.52)	7.18	(6.77–7.61)	0.41	11.6	27.7	0.85	(0.78–0.90)
** SM (OH) C16:1**	3.57	(3.37–3.78)	3.56	(3.35–3.79)	0.95	13.7	26.3	0.79	(0.70–0.85)
** SM (OH) C22:1**	13.7	(13.1–14.5)	13.9	(13.2–14.5)	0.69	13.7	20.7	0.70	(0.58–0.78)
** SM (OH) C22:2**	11.6	(10.9–12.3)	11.6	(11.0–12.3)	0.83	13.1	25.5	0.79	(0.70–0.85)
** SM (OH) C24:1**	1.26	(1.19–1.34)	1.22	(1.15–1.30	0.26	18.9	24.6	0.63	(0.49–0.73)
** SM C16:0**	125	(120–130)	127	(122–132)	0.42	11.3	15.5	0.66	(0.53–0.75)
** SM C16:1**	17.9	(17.0–18.7)	18.1	(17.4–19.0)	0.33	11.4	19.8	0.75	(0.65–0.83)
** SM C18:0**	26.4	(25.3–27.6)	26.7	(25.5–27.9)	0.60	12.8	19.0	0.69	(0.57–0.78)
** SM C18:1**	11.50	(10.9–12.1)	11.5	(10.9–12.1)	0.98	12.7	23.4	0.77	(0.68–0.84)
** SM C20:2**	0.46	(0.42–0.50)	0.44	(0.41–0.47)	0.39	32.3	19.1	0.26	(0.07–0.43)
** SM C22:3**	1.99	(1.81–2.19)	2.23	(2.08–2.38)	0.03	34.2	22.5	0.30	(0.11–0.47)
** SM C24:0**	24.0	(22.9–25.1)	24.1	(23.1–25.1)	0.78	14.2	16.3	0.57	(0.42–0.69)
** SM C24:1**	51.9	(49.8–54.2)	52.3	(50.0–54.6)	0.73	12.6	17.5	0.66	(0.54–0.76)
** SM C26:0**	0.21	(0.20–0.22)	0.22	(0.20–0.23)	0.48	29.0	16.2	0.24	(0.04–0.41)
** SM C26:1**	0.46	(0.44–0.49)	0.43	(0.40–0.46)	0.04	24.4	19.8	0.40	(0.22–0.55)
**Hexose**	5059	(4811–5320)	5207	(4940–5488)	0.11	12.7	22.6	0.76	(0.66–0.83)

a Abbreviations are as follows: Cx:y (x = number of carbons in the fatty acid side chain, y  =  number of double bonds in the fatty acid side chain), hydroxyl (OH), phosphatidylcholine (PC), acyl (a), sphingomyelin (SM). For detailed nomenclature see Table S1.

b n = 100; except: lysoPC a C6:0 n = 61; SM C20:2 and SM C22:3 n = 99.

c A paired t-test based on log-transformed values was calculated to compare geometric means of metabolite concentrations over-time.

d Based on log transformed values.

e ICC calculated as the ratio of between-person variance and total variance.

f For negative values ICC was calculated based on positive variance estimators [33].

g Metabolite concentration was below the assay's limit of detection.

**Table 4 pone-0021103-t004:** Geometric Means, Variances and Intraclass-Correlation Coefficients (ICCs) of Serum Concentrations of Diacyl-Glycerophospholipids Measured 4 Months Apart Among 100 Healthy Subjects from the EPIC-Potsdam Cohort.

	1st Measurement		2nd Measurement			CV (%)			
Metabolite [µmol/L]^a^ ^b^	Mean	95% CI	Mean	95% CI	*P-value* c	Within-person	Between-person	ICC^d^^,^ e ^,^ f	95% CI
**PC aa C24:0**	0.12	(0.11–0.13)	0.12	(0.11–0.13)	0.56	32.3	25.8	0.39	(0.21–0.54)
**PC aa C26:0** g	0.84	(0.79–0.89)	0.80	(0.77–0.84)	0.04	16.6	20.4	0.60	(0.46–0.71)
**PC aa C28:1**	3.55	(3.37–3.74)	3.59	(3.43–3.77)	0.51	13.4	21.3	0.72	(0.61–0.80)
**PC aa C30:0**	4.89	(4.62–5.18)	4.86	(4.58–5.16)	0.81	19.6	21.8	0.55	(0.40–0.67)
**PC aa C30:2**	0.12	(0.10–0.16)	0.13	(0.10–0.16)	0.30	96.6	51.9	0.27	(0.00–0.43)
**PC aa C32:0**	16.2	(15.6–16.8)	16.4	(15.8–17.1)	0.38	13.2	14.3	0.54	(0.39–0.66)
**PC aa C32:1**	19.3	(17.6–21.2)	19.2	(17.4–21.2)	0.88	26.4	40.3	0.70	(0.58–0.79)
**PC aa C32:2**	4.71	(4.39–5.05)	4.59	(4.26-4.96)	0.44	23.6	28.2	0.59	(0.44–0.70)
**PC aa C32:3**	0.54	(0.51–0.57)	0.55	(0.52–0.58)	0.35	16.2	21.9	0.65	(0.52–0.75)
**PC aa C34:1**	245	(233–259)	254	(242–267)	0.13	16.2	20.3	0.61	(0.47–0.72)
**PC aa C34:2**	451	(433–469)	466	(450–483)	0.07	12.6	14.1	0.55	(0.40–0.68)
**PC aa C34:3**	17.8	(16.9–18.9)	18.0	(16.9–19.0)	0.80	18.8	21.8	0.57	(0.43–0.69)
**PC aa C34:4**	2.06	(1.92–2.21)	2.06	(1.92–2.22)	0.96	22.9	27.7	0.59	(0.45–0.71)
**PC aa C36:0**	2.72	(2.58–2.86)	2.79	(2.64–2.94)	0.30	17.8	20.1	0.56	(0.41–0.68)
**PC aa C36:1**	53.1	(50.4–55.8)	53.7	(51.3–56.1)	0.61	16.1	17.7	0.55	(0.39–0.67)
**PC aa C36:2**	267	(256–278)	272	(262–283)	0.32	14.3	13.2	0.46	(0.29–0.60)
**PC aa C36:3**	142	(135–148)	144	(138–150)	0.52	15.4	15.8	0.51	(0.35–0.64)
**PC aa C36:4**	200	(191–211)	209	(199–220)	0.02	13.5	20.8	0.70	(0.59–0.79)
**PC aa C36:5**	30.6	(27.9–33.6)	29.0	(26.5–31.8)	0.21	29.9	35.4	0.58	(0.44–0.70)
**PC aa C36:6**	1.16	(1.07–1.24)	1.09	(1.01–1.18)	0.11	24.6	28.9	0.58	(0.43–0.70)
**PC aa C38:0**	2.91	(2.75–3.08)	2.93	(2.78–3.10)	0.76	16.2	22.9	0.67	(0.540.76)
**PC aa C38:1**	0.43	(0.34–0.55)	0.43	(0.35–0.52)	0.94	100.5	15.3	0.03	(0.00–0.23)
**PC aa C38:3**	53.6	(50.9–56.4)	53.5	(51.1–56.0)	0.94	16.9	17.7	0.52	(0.37–0.65)
**PC aa C38:4**	118	(112–125)	123	(116–130)	0.08	14.6	22.6	0.70	(0.59–0.79)
**PC aa C38:5**	59.0	(56.0–62.2)	58.4	(55.4–61.6)	0.65	15.9	21.4	0.64	(0.51–0.75)
**PC aa C38:6**	86.0	(81.2–91.0)	84.9	(80.1-90.1)	0.61	16.8	23.9	0.67	(0.55–0.77)
**PC aa C40:1** g	0.42	(0.40–0.44)	0.40	(0.38–0.43)	0.26	18.3	17.2	0.47	(0.30–0.61)
**PC aa C40:2**	0.26	(0.24–0.28)	0.25	(0.23–0.27)	0.23	35.4	14.3	0.14	(0.00–0.33)
**PC aa C40:3**	0.48	(0.45–0.51)	0.46	(0.44–0.49)	0.28	26.9	16.5	0.28	(0.08–0.45)
**PC aa C40:4**	3.74	(3.54–3.94)	3.88	(3.68–4.09)	0.14	17.7	20.4	0.57	(0.42–0.69)
**PC aa C40:5**	10.7	(10.1–11.3)	10.7	(10.1–11.3)	0.90	17.4	22.4	0.62	(0.49–0.73)
**PC aa C40:6**	31.0	(29.1–33.1)	30.4	(28.5–32.4)	0.48	19.4	25.8	0.64	(0.51–0.74)
**PC aa C42:0**	0.54	(0.50–0.57)	0.53	(0.50–0.57)	0.98	17.2	28.7	0.73	(0.63–0.81)
**PC aa C42:1**	0.25	(0.24–0.27)	0.26	(0.24–0.28)	0.59	19.8	28.1	0.67	(0.54–0.76)
**PC aa C42:2**	0.21	(0.20–0.22)	0.21	(0.20–0.22)	0.87	22.8	18.8	0.40	(0.23-0.56)
**PC aa C42:4**	0.17	(0.16–0.17)	0.17	(0.16–0.18)	0.29	24.5	11.4	0.18	(0.00–0.36)
**PC aa C42:5**	0.39	(0.37–0.42)	0.37	(0.35–0.39)	0.13	23.2	19.7	0.42	(0.24-0.57)
**PC aa C42:6**	0.65	(0.63–0.68)	0.63	(0.60–0.66)	0.09	15.2	15.7	0.52	(0.36–0.65)

a Abbreviations are as follows: Cx:y (x = number of carbons in the fatty acid side chain, y  =  number of double bonds in the fatty acid side chain), phosphatidylcholine (PC), acyl-acyl (aa). For detailed nomenclature see Table S1.

b n = 100; except: PC aa C30:2 n = 73; PC aa C38:1 n = 82.

c A paired t-test based on log-transformed values was calculated to compare geometric means of metabolite concentrations over-time.

d Based on log transformed values.

e ICC calculated as the ratio of between-person variance and total variance.

f For negative values ICC was calculated based on positive variance estimators [33].

g Metabolite concentration was below the assay's limit of detection.

**Table 5:** Geometric Means, Variances and Intraclass-Correlation Coefficients (ICCs) of Serum Concentrations of Acyl-Alkyl-Glycerophospholipids Measured 4 Months Apart Among 100 Healthy Subjects from the EPIC-Potsdam Cohort.

	1st Measurement		2nd Measurement			CV (%)			
Metabolite [µmol/L]^a^ ^b^	Mean	95% CI	Mean	95% CI	*P-value* c	Within-person	Between-person	ICC^d^^,^ e ^,^ f	95% CI
**PC ae C30:0**	0.38	(0.36–0.40)	0.38	(0.36–0.41)	0.96	18.2	24.6	0.65	(0.52–0.75)
**PC ae C30:1**	0.25	(0.22–0.28)	0.21	(0.18–0.25)	0.12	67.9	17.7	0.06	(0.00–0.26)
**PC ae C30:2**	0.11	(0.11–0.12)	0.11	(0.10–0.12)	0.40	22.8	23.9	0.52	(0.37–0.65)
**PC ae C32:1**	3.07	(2.94–3.21)	3.10	(2.96–3.25)	0.64	13.3	18.4	0.66	(0.53–0.75)
**PC ae C32:2**	0.79	(0.75–0.83)	0.78	(0.75–0.82)	0.71	14.0	21.1	0.70	(0.58–0.78)
**PC ae C34:0**	1.55	(1.47-1.63)	1.57	(1.48–1.66)	0.70	18.0	20.9	0.57	(0.43–0.69)
**PC ae C34:1**	10.8	(10.4–11.3)	11.0	(10.5–11.5)	0.42	14.1	17.7	0.61	(0.47–0.72)
**PC ae C34:2**	12.8	(12.1–13.5)	13.2	(12.5–14.0)	0.14	17.1	22.3	0.63	(0.49–0.73)
**PC ae C34:3**	7.98	(7.54–8.44)	8.41	(7.93–8.91)	0.03	16.7	23.7	0.67	(0.54–0.76)
**PC ae C36:0**	0.68	(0.65–0.72)	0.71	(0.67–0.74)	0.18	17.4	20.1	0.57	(0.42–0.69)
**PC ae C36:1**	8.81	(8.40–9.23)	8.84	(8.44–9.27)	0.84	14.0	19.2	0.65	(0.53–0.75)
**PC ae C36:2**	14.1	(13.4–14.9)	14.1	(13.4–14.9)	1.00	13.0	23.0	0.76	(0.66–0.83)
**PC ae C36:3**	7.79	(7.39–8.21)	8.14	(7.74–8.55)	0.10	18.6	17.9	0.48	(0.32–0.62)
**PC ae C36:4**	18.8	(17.8–19.8)	19.9	(19.0–21.0)	0.02	17.8	19.4	0.54	(0.39–0.67)
**PC ae C36:5**	12.0	(11.3–12.6)	12.6	(11.9–13.3)	0.03	16.1	21.8	0.65	(0.52–0.75)
**PC ae C38:0**	1.69	(1.60–1.78)	1.66	(1.57–1.76)	0.49	17.3	21.6	0.61	(0.47–0.72)
**PC ae C38:1**	0.67	(0.60–0.74)	0.75	(0.69–0.82)	0.06	46.1	16.1	0.11	(0.00–0.30)
**PC ae C38:2**	1.48	(1.40–1.56)	1.44	(1.35–1.53)	0.38	24.5	15.7	0.29	(0.10–0.46)
**PC ae C38:3**	4.23	(4.03–4.45)	4.24	(4.04–4.45)	0.95	14.9	19.4	0.63	(0.50–0.73)
**PC ae C38:4**	13.5	(12.9–14.1)	14.0	(13.4–14.7)	0.04	13.5	18.6	0.65	(0.53–0.75)
**PC ae C38:5**	17.6	(16.8–18.4)	18.4	(17.6–19.2)	0.03	14.4	18.1	0.61	(0.47–0.72)
**PC ae C38:6**	7.82	(7.40–8.26)	7.87	(7.47–8.29)	0.76	16.2	21.6	0.64	(0.51–0.74)
**PC ae C40:0** g	8.62	(8.36–8.89)	8.59	(8.33–8.85)	0.80	10.3	11.5	0.56	(0.40–0.68)
**PC ae C40:1**	1.04	(0.98–1.09)	1.03	(0.98–1.09)	0.95	17.3	20.7	0.59	(0.44–0.70)
**PC ae C40:2**	2.12	(2.02–2.22)	2.11	(2.00–2.24)	0.94	14.0	22.5	0.72	(0.61–0.80)
**PC ae C40:3**	1.05	(1.00–1.10)	1.01	(0.96–1.06)	0.07	15.2	18.4	0.60	(0.45–0.71)
**PC ae C40:4**	2.11	(2.02–2.21)	2.18	(2.07–2.29	0.12	13.6	20.6	0.70	(0.58–0.79)
**PC ae C40:5**	3.66	(3.52–3.81)	3.71	(3.54–3.89)	0.51	14.0	17.1	0.60	(0.46–0.71)
**PC ae C40:6**	4.81	(4.59–5.05)	4.79	(4.55–5.05)	0.80	13.9	20.8	0.69	(0.57-0.78)
**PC ae C42:0** g	0.31	(0.30–0.32)	0.30	(0.29–0.32)	0.25	18.4	5.5	0.08	(0.00–0.27)
**PC ae C42:1**	0.36	(0.34–0.38)	0.36	(0.34–0.38)	0.93	19.0	18.7	0.49	(0.33-0.63)
**PC ae C42:2**	0.64	(0.61–0.67)	0.63	(0.60–0.66)	0.35	16.1	18.4	0.56	(0.42–0.68)
**PC ae C42:3**	0.68	(0.65–0.72)	0.65	(0.61–0.69)	0.10	18.8	22.3	0.59	(0.44–0.70)
**PC ae C42:4**	0.93	(0.88–0.98)	0.95	(0.90–1.01)	0.26	13.8	24.2	0.75	(0.65–0.83)
**PC ae C42:5**	2.10	(2.00–2.20)	2.12	(2.01–2.22)	0.66	11.7	21.5	0.77	(0.68–0.84)
**PC ae C44:3**	0.11	(0.10–0.12)	0.10	(0.10–0.11)	0.06	25.0	23.5	0.47	(0.30–0.61)
**PC ae C44:4**	0.38	(0.36–0.41)	0.40	(0.37–0.43)	0.10	18.8	29.1	0.71	(0.59–0.79)
**PC ae C44:5**	1.91	(1.81–2.02)	1.94	(1.83–2.06)	0.45	15.0	25.3	0.74	(0.64–0.82)
**PC ae C44:6**	1.27	(1.20–1.35)	1.30	(1.22–1.38)	0.38	13.3	27.3	0.81	(0.73–0.87)

a Abbreviations are as follows: Cx:y (x = number of carbons in the fatty acid side chain, y  =  number of double bonds in the fatty acid side chain), phosphatidylcholine (PC), acyl-alkyl (ae). For detailed nomenclature see Table S1.

b n = 100; except: PC ae C30:1 n = 97.

c A paired t-test based on log-transformed values was calculated to compare geometric means of metabolite concentrations over-time.

d Based on log transformed values.

e ICC calculated as the ratio of between-person variance and total variance.

f For negative values ICC was calculated based on positive variance estimators [33].

g Metabolite concentration was below the assay's limit of detection.

**Figure 1:**
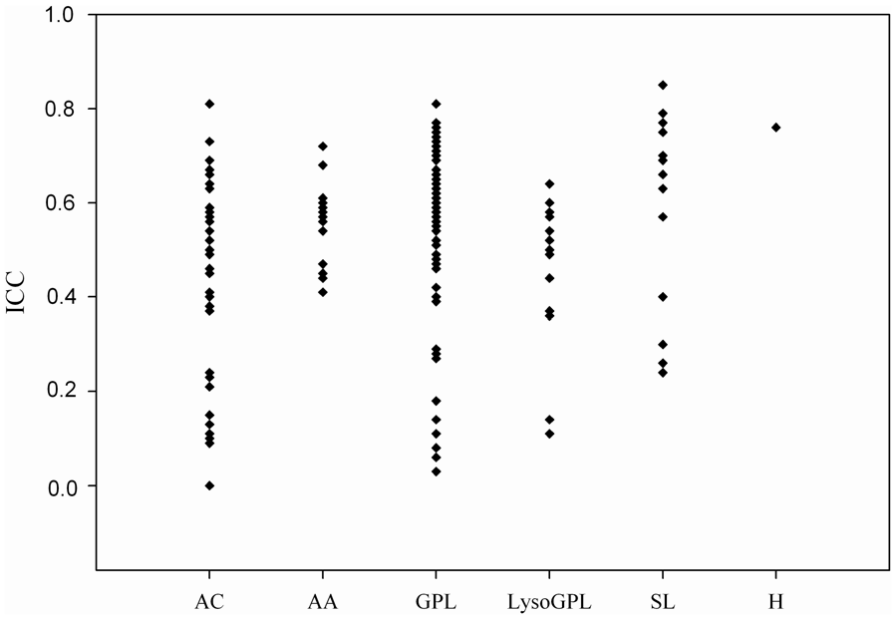
Intraclass-Correlation Coefficients (ICCs) of 163 Serum Metabolites According to Metabolite Subclass Measured 4 Months Apart. Each data point represents the ICC of one metabolite among 100 healthy subjects from the EPIC-Potsdam cohort (n = 100; except: PC aa C30:2 n = 73; PC aa C38:1 n = 82; PC ae C30:1 n = 97; lysoPC a C6:0 n = 61; SM C20:2 and SM C22:3 n = 99). Abbreviations: AC: acylcarnitines, AA: amino acids, GPL: glycerophospholipids, lysoGPL: lysoglycerophospholipids, SL: sphingolipids, H: hexose.

Among metabolite subclasses (**Figure 1**), the serum reliability was excellent in hexose (ICC = 0.76), the 15 sphingolipids showed a poor to excellent reliability (median ICC = 0.66, range: 0.24–0.85), reliability of the 14 amino acids was fair to good with a median ICC of 0.58 (range: 0.41–0.72) and the 92 glycerophospholipids had a poor to excellent reliability with median ICC = 0.58 (range: 0.03–0.81). The least reliable metabolite subclass included the 41 acylcarnitines with median ICC = 0.45 (range: 0.00–0.81). However, acylcarnitines were a very heterogeneous class. Among acylcarnitines, most of the short and medium chain saturated compounds showed a good to excellent reliability (ICC ranging from 0.50–0.81), whereas reliability of hydroxyacylcarnitines and monounsaturated acylcarnitines was mostly poor to fair (ICC range: 0.11–0.45 and 0.00–0.63, respectively). The analytical variance of the metabolites was also evaluated measuring 230 replicates (**Tables S1** and **S2**). 29 of the metabolites, most of them monounsaturated- and hydroxyacylcarnitines, next to few glycerophospholipids, showed very low serum concentrations that were below the limit of detection (LOD) of the analytical method. After excluding these metabolites from the ICC calculation, the overall corrected median ICC_corr_ was 0.59, and in specific for acylcarnitines median ICC_corr_ was 0.52 and for glycerophospholipids median ICC_corr_ was 0.59.

Since reliability depends on both, within- and between-person variances, we also report these variance components in **Tables 2** to [Table pone-0021103-t003] [Table pone-0021103-t004] **5** **,** to illustrate their contribution to the ICC. For example, the acylcarnitines C4 and C16 had very similar within-person variances (18.7% vs. 18.8%, respectively); however, their ICCs were different (0.49 vs. 0.81, respectively) (**Table 2**), which was explained by their differing between-person variances (18.3% vs. 38.4%, respectively). Acylcarnitine C4 showed a much lower within-person variance compared to its between-person variance (18.8% vs. 38.4%, respectively), leading to a high ICC (0.81) and indicating excellent reliability; whereas acylcarnitine C16 showed similar within- and between-person variances (18.7% vs. 18.3%, respectively), leading to a lower ICC (0.49) and suggesting only fair reliability.

## Discussion

This study, investigating the reliability of 163 serum metabolites under fasting conditions using a commercially available kit in a healthy sub-sample of the population-based EPIC-Potsdam cohort over a 4-month period, observed an acceptable reliability for most of the metabolites. Reliability was fair to excellent for hexose, all of the amino acids, saturated short- and medium chain acylcarnitines and most of the sphingomyelins and glycerophospholipids. The results support the concept that these metabolites are reliable candidates for risk assessment in prospective epidemiologic studies with one blood sample collection, as a single measurement appropriately reflects their long-term concentration in individuals. Few compounds revealed higher variability, such as hydroxyacylcarnitines and monounsaturated acylcarnitines, which imply restrain conditions in the design of epidemiologic studies.

A single assessment of a biochemical indicator may be susceptible to short-term variation and not reflect true long-term exposure. The ICC, as the ratio of between-person variance and total variance (sum of between- and within-person variances), reflects reliability very well as it considers both between- and within-person variability. A high ICC can be obtained by low within- and/or high between-person variance. A low ICC is attributable to high within- and/or low between-person variance. Random measurement error generally tends to decrease correlation and regression coefficients in epidemiologic studies toward 0 and bias relative risks toward 1. To assess long-term exposure using a single blood measurement, as it is often the case in large epidemiologic studies, the between-person variance should account for most of the observed variability in the biomarker concentration, whereas within-person variance should be relatively low [5], [9]. This requirement was met by the majority of the metabolites that were included in the present study.

Reliability studies often focus on technological improvement of biochemical assays, sample handling or storage conditions [10], [11], [12], [13]. However, studies investigating the biological variance and over-time reliability of metabolite concentrations are rarely found in the literature [14], [15], [16]. Previous studies focused on biological variation of metabolites in other biological fluids e.g. plasma or cerebral spinal fluid, were based on a limited number of samples or used an untargeted metabolomics strategy. Investigators previously reported similar high ICCs for plasma concentrations of amino acids in different nutritional states [17]. Amino acid metabolism is tightly regulated, and a genetic component has been suggested to play a major role in amino acid homeostasis [17]. Therefore, intra-individual blood concentrations of amino acids are within a narrow range. This fact is also reflected in our results, as we found that within-person variance in amino acids is in a lower range than between-person variance.

We found low within-person variance and excellent reliability of hexose concentrations. Hexose includes various monosaccharides containing 6 carbons, e.g. glucose, fructose and galactose, among others. Hormonal control mechanisms immediately respond to feeding, postprandial and fasting states and ensure that blood sugar concentrations are contained within a narrow range over-time.

Acylcarnitines represent esterified fatty acid derivates that occur in the process of fatty acid translocation into the inner mitochondrial membrane which is the limiting step for β-oxidation. Acylcarnitines can efficiently pass into the cytosol and subsequently into the blood stream. Blood acylcarnitine concentrations, therefore, reflect the substrate flux through β-oxidation. Increased acylcarnitine concentrations have been associated with type 2 diabetes mellitus previously [18], [19]. However, information on serum reliability is scarce. We found that short and medium chain acylcarnitines in serum are more reliable than longer chain hydroxy- and monounsaturated acylcarnitines. In healthy human individuals, acylcarnitines are generally observed at low concentrations in plasma and serum. This might also affect the observed ICCs and the analytical variance. Per example, all hydroxylated acylcarnitines were below the LOD, except C14:1-OH, which showed values very close to the LOD. Therefore, the analytical variance observed for these metabolites is presented in **Table S2**.

Phosphatidylcholines belong to the group of membrane phospholipids that consist of a glycerol core which carries a choline head group and two fatty acid residues. Lysophosphatidylcholines usually originate from hydrolysis of the sn-2 fatty acid and transesterification by phospholipase A2 and, therefore, only carry one fatty acid. De-novo synthesis and redistribution from plasma membranes may impact phosphatidylcholine and lysophosphatidylcholine concentrations in blood [20]. Sphingomyelins are also membrane phospholipids, but instead of glycerol, they contain a ceramide core, including a fatty acid, and a polar head group. Besides being part of membranes, they are also involved in signal transduction such as nuclear factor-κB pathways [21]. This action requires enzymatical breakdown of sphingomyelins and release of ceramides [22]. A previous study that investigated the reproducibility of platelet phospholipid measures in 12 subjects over a 3-week period reported similar ICCs as observed in our study [23]. That study found an ICC of 0.50 for total phosphatidylcholines and an ICC of 0.54 for total sphingomyelins, as compared to a median ICC of 0.58 for phosphatidylcholines and median ICC of 0.66 for sphingomyelins observed in our study. These findings indicate good reliability of most phospholipids and support their usefulness as reliable candidate biomarkers.

The strength of our study was that we assessed reliability in a wide spectrum of metabolites including different classes of compounds. Furthermore, the detection assay for these metabolites represents a modern high-throughput technique that has already been approved and standardized, and can be applied to future metabolome analysis. We also used a sub-sample of a large population-based prospective cohort study for this reliability investigation, therefore, ensuring a high precision for the results of the ICCs. The participants of this study were free-living, and thus, their exposure to various external factors differed reflecting a real life situation.

Our study had some limitations. The estimation of within-person variation (% CV) was based on only two time point measurements. This was a trade-off for the large sample size of the present study. To account for this limitation we included a long time span between the two measurements of metabolite concentrations where participants were free-living and thus, exposed to several external factors that could have affected stability of metabolite concentrations. The present study included fasting healthy subjects. Reliability of metabolites may be different for people with existing chronic disease and in situations in which fasting status is not possible to obtain. Future studies are warranted to further investigate the metabolite reliability in different (i.e. those with overt disease or challenged) populations.

We were primarily interested to study the reliability of metabolites for risk assessment using a single blood measurement, as is usually the case in large scale epidemiologic studies. Therefore, we did not investigate in detail possible sources of within- or between-person variance. We are aware of a need to evaluate the impact of genetic and non-genetic factors on these variance components, which should be the aim of future studies. Some metabolites, especially of the acylcarnitine group, showed lower serum concentrations than the LOD of the assay system; thus, the observed low ICCs could also be explained by technological limitation. Beyond biologic variability, the total variance of the biomarker concentrations depends on the precision of the measurement. Although most metabolites were measured with relatively high precision, the coefficients of variations were larger for a few metabolites, which may explain the lower reliability for some of these markers. Thus, the reliability depends on the assay system, and, although we used a validated kit, the reliability of the metabolites measured here may be different when other systems are used. Although reliability was low for some of the metabolic markers when analysed separately as in our analysis, this does not exclude the possibility that these markers may still be useful when investigating the impact of metabolic profiles on disease risk.

In conclusion, we found fair to excellent reliability for most of the metabolites, including short- and medium chain acylcarnitines, amino acids, hexose and phospholipids in free-living healthy subjects. Our results suggest that a single assessment of these metabolites may be sufficient for risk assessment in prospective epidemiologic studies. In contrast, reliability was lower for monounsaturated- and hydroxyl-acylcarnitines and few glycerophospholipids, which is most likely explained by their low serum concentrations in healthy individuals that exceed the assay's detection limit at the present time. This may limit their use for risk assessment when based on a single measurement.

## Materials and Methods

### Ethics statement

All of the EPIC-Potsdam participants gave written informed consent and the study was approved by the ethics committee of the medical association of the state of Brandenburg, Germany.

### Study population

The EPIC-Potsdam cohort was recruited from the general population and consists of 27,548 participants mainly aged 35–65 years at time of recruitment between 1994 and 1998 [24], [25], [26]. In 2007, a group of 407 EPIC-Potsdam participants were invited to participate in a validation study of physical activity assessment within the EPIC study, which included the collection of two blood collections approximately 4 month apart in time. The subjects were randomly selected among all EPIC participants who were younger than 64 years, had a valid telephone number, had residence within a 5 km radius of the study center, and with systolic blood pressure <180 mmHg, and diastolic blood pressure <110 mmHg at time of recruitment. Exclusion criteria were a history of heart disease (myocardial infarct, heart failure, cardiomyopathy, stroke or angina pectoris), use of β–blockers, or impaired mobility, as documented in the EPIC database at the time of the invitation. Of the 407 subjects, 11 did not respond, 176 declined participation and 12 were excluded after a phone interview with the study physician had revealed β-blockers medication. Thus, a total of 208 EPIC-Potsdam subjects (83 men and 125 women) participated in the validation study of physical activity assessment. Out of this sub-sample, a total of 100 subjects (50 men and 50 women) were randomly selected among those who had provided two fasting blood samples over a period of 4 month. The first blood withdrawal was conducted between October 2007 and March 2008 and the second blood sample was collected between February 2008 and July 2008. Fasting blood was drawn by qualified medical staff in a standardized procedure using monovette tubes with coagulation activator. Serum was fractionated by centrifugation at 2,700 xg for 10 minutes, and stored in a freezer at −80°C until analysis.

### Serum metabolite concentrations

Serum concentrations of 163 metabolites were determined using a targeted metabolomic approach with the Absolute*IDQ*™ p150 kit (BIOCRATES Life Sciences AG, Innsbruck, Austria). The samples were prepared according the manufacturers protocol and the assay procedures have been described in our previous work [27]. In short: After centrifugation, 10 µL of serum were pipetted onto a inserted filter in a 96 well sandwich plate, which already contained stable isotope labeled internal standards. The filters were dried in nitrogen stream, amino acids were derivated with 5% phenylisothiocyanate reagent (PITC) and filters were dried again. After extraction of metabolites and internal standards with 5 mM ammonium acetate in methanol, the solution was centrifuged through the filter membrane and diluted with MS running solvent. Final extracts were analyzed by FIA-MS/MS. Detailed description of the procedure has been described previously [27]. Metabolites were quantified by reference to appropriate internal standards. The method was proven to be in conformance with FDA-Guidline “Guidance for Industry - Bioanalytical Method Validation (May 2001), which implies proof of reproducibility within a given error range. Measurements were performed as described in the Biocrates user's manual UM-P150. Analytical specifications for the LOD, evaluated quantification ranges, further LOD for semiquantitative measurements, identities of quantitative and semiquantitative metabolites, specificity, potential interferences, linearity, precision and accuracy, reproducibility and stability were described in BIOCRATES manual AS-P150. The LODs were set to three times the values of zero samples (PBS with internal standards). The lower limit of quantification (LLOQ) and upper limit of quantification (ULOQ) were determined experimentally by BIOCRATES. This information is provided in **Table S1**. The median analytical variance was 7.3% within-plate CV and 11.4% between-plate CV. It was determined by measuring 230 replicates (46 plates each containing 5 replicates) of one female serum sample and is reported in detail in **Table S2**. The Absolute*IDQ*™ p15 kit has previously been applied in metabolomic studies in humans (7, 8,).

### Metabolomic platform

The metabolomic measurements allowed simultaneous quantification of 163 metabolites including 41 acylcarnitines (Cx:y), 14 amino acids, 1 hexose, 92 glycerophospholipids (lysophosphatidylcholines (lysoPC), diacyl- and acyl-alkyl- phosphatidylcholines (PC)), and 15 sphingolipids (SMx:y) in a one-step analysis.

The detailed nomenclature is provided in **Table S1**. Lipid side chains were abbreviated as Cx:y, where x equaled the number of carbons in the side chain and y denoted the number of double bonds. The technology was limited as it could not detect the distribution of the carbon atoms among 146 different fatty acids and the exact position of the double bonds in complex lipids. All glycerophospholipids were phosphatidylcholines (PC), that were further differentiated with respect to ester (a) and ether (e) bonds, where two letters implied that two fatty acids are bond to glycerol (aa = diacyl, ae = acyl-alkyl), while one letter (a = acyl, e = alkyl) and the prefix ‘lyso’ indicated the presence of a single fatty acid residue; e.g. lysoPCaC24:0  = lysophosphatidylcholine acyl C24:0 (lignoceric acid). Sphingolipids were sphingomyelins (SM) and hydroxysphingomyelins (SM(OH)). All acylcarnitines were naturally occurring L-isomers abbreviated according to the fatty acid that was bond (e.g. C2 = acetyl-L-carnitine). DL-carnitine was abbreviated as C0. Amino acids were presented according to standard three letter abbreviations.

A substantial part of the metabolites determined by Absolute*IDQ*™ p150 kit showed values below LOD. However, it was important to measure those as well, as their concentrations may increase drastically in disease or upon environmental challenge [28], [29], [30]. Therefore, metabolites with a concentration lower than the LOD (n = 29) were further reported but labeled accordingly in the tables.

### Statistical analysis

The metabolite serum concentrations were not normally distributed as indicated by Kolmogorov-Smirnov test, but right-skewed. Therefore, the concentrations were log-transformed and reported as geometric means and 95% confidence intervals (CIs). Student's paired t-test was used to compare the concentrations of each metabolite measured 4 month apart. Variance components were estimated with a one-way random effects model and subject ID as the random variable (SAS procedure: PROC ANOVA) [5]. The between- and within-person CVs (biological variance) as well as the between- and within-plate CVs (analytical variance) were calculated as the square root of the between- and within-person/plate variance components from the random effects model on a log-transformed scale [31]. To assess reliability of serum metabolite concentrations, we calculated ICCs by dividing the between-person variance by the total variance (sum of between- and within-person variances), and calculated 95% CI of ICCs [32]. For negative values, ICCs were calculated based on positive variance estimators [33]. An ICC ≥0.75 was considered to indicate excellent reliability; ICCs between 0.51 and 0.74 to indicate good reliability; ICCs between 0.40 and 0.50 to indicate fair reliability and an ICC <0.40 was considered as poorly reliable (16). All statistical analyses were performed with SAS software, release 9.2, (SAS Institute Inc., Cary, NC). The level of statistical significance was set at *P*<0.05 for two-sided testing.

## Supporting Information

Table S1 **Biochemical Names and Quantification Ranges of 163 Metabolites Measured with the BIOCRATES Absolute IDQ Targeted Metabolomics Technology.** Footnote: Abbreviations: LOD, limit of detection; LLOQ, lower limit of quantification; ULOQ, upper limit of quantification. ^a^The quantification range was determined by BIOCRATES and adopted from the Manual: “Absolute*IDQ*™ p150 kit – Analytical Specifications” (BIOCRATES Life Sciences AG, Innsbruck, Austria).
(DOC)

Table S2 **Analytical Variance of 163 Metabolites Measured with the BIOCRATES Absolute IDQ Targeted Metabolomics Technology.** Footnote: Abbreviations: CV, coefficient of variation; LOD, limit of detection. ^a^Analytical variance was determined by measuring 5 replicates on each of the 46 plates containing the EPIC-samples (total of 230 replicates) and reported as CV%. Note: higher CVs are mainly observed for metabolites that show very low concentrations and are below the LOD of the assay. ^b^Metabolite concentration was below the assay's LOD.
(DOCX)
