# Supplementary material for: Reliability of Serum Metabolite Concentrations over a 4-Month Period Using a Targeted Metabolomic Approach
Source: PLoS One. 2011 Jun 15;6(6):e21103. doi: 10.1371/journal.pone.0021103 (PMC3115978; doi:10.1371/journal.pone.0021103)
Supplement: Table S1 — Biochemical Names and Quantification Ranges of 163 Metabolites Measured with the BIOCRATES Absolute IDQ Targeted Metabolomics Technology. Footnote: Abbreviations: LOD, limit of detection; LLOQ, lower limit of quantification; ULOQ, upper limit of quantification. aThe quantification range was determined by BIOCRATES and adopted from the Manual: “AbsoluteIDQ™ p150 kit – Analytical Specifications” (BIOCRATES Life Sciences AG, Innsbruck, Austria). (DOC) [file pone.0021103.s001.doc]

| **Table S1: Biochemical names and quantification ranges of 163 metabolites measured with the BIOCRATES Absolute IDQ targeted metabolomics technology** | | | | |
| --- | --- | --- | --- | --- |
| ***Abbreviation*** | ***Biochemical name*** | ***Quantification Range^a^*** | | |
|  |  | ***LOD***  ***(µM)*** | ***LLOQ***  ***(µM)*** | ***ULOQ***  ***(µM)*** |
| Acylcarnitines |  |  |  |  |
| C0 | DL-Carnitine | 4 | 5 | 120 |
| C10 | Decanoyl-L-carnitine | 0.16 | 0.3 | 6 |
| C10:1 | Decenoyl-L-carnitine | 0.12 |  |  |
| C10:2 | Decadienyl-L-carnitine | 0.04 |  |  |
| C12 | Dodecanoyl-L-carnitine | 0.057 | 0.4 | 12 |
| C12-DC | Dodecanedioyl-L-carnitine | 0.2 |  |  |
| C12:1 | Dodecenoyl-L-carnitine | 0.2 |  |  |
| C14 | Tetradecanoyl-L-carnitine | 0.03 | 0.4 | 6 |
| C14:1 | Tetradecenoyl-L-carnitine | 0.015 |  |  |
| C14:1-OH | Hydroxytetradecenoyl-L-carnitine | 0.015 |  |  |
| C14:2 | Tetradecadienyl-L-carnitine | 0.012 |  |  |
| C14:2-OH | Hydroxytetradecadienyl-L-carnitine | 0.015 |  |  |
| C16 | Hexadecanoyl-L-carnitine | 0.018 | 0.4 | 12 |
| C16-OH | Hydroxyhexadecanoyl-L-carnitine | 0.015 |  |  |
| C16:1 | Hexadecenoyl-L-carnitine | 0.06 |  |  |
| C16:1-OH | Hydroxyhexadecenoyl-L-carnitine | 0.02 |  |  |
| C16:2 | Hexadecadienyl-L-carnitine | 0.008 |  |  |
| C16:2-OH | Hydroxyhexadecadienyl-L-carnitine | 0.03 |  |  |
| C18 | Octadecanoyl-L-carnitine | 0.02 | 0.4 | 6 |
| C18:1 | Octadecenoyl-L-carnitine | 0.04 |  |  |
| C18:1-OH | Hydroxyoctadecenoyl-L-carnitine | 0.023 |  |  |
| C18:2 | Octadecadienyl-L-carnitine | 0.009 |  |  |
| C2 | Acetyl-L-carnitine | 0.15 | 0.4 | 12 |
| C3 | Propionyl-L-carnitine | 0.08 | 0.4 | 15 |
| C3-DC / C4-OH | Malonyl-L-carnitine / Hydroxybutyryl-L-carnitine | 0.09 |  |  |
| C3-DC-M / C5-OH | Methylmalonyl-L-carnitine / Hydroxyvaleryl-L-carnitine | 0.1 |  |  |
| C3-OH | Hydroxypropionyl-L-carnitine | 0.05 |  |  |
| C3:1 | Propenyl-L-carnitine | 0.03 |  |  |
| C4 | Butyryl-L-carnitine | 0.03 | 0.4 | 12 |
| C4:1 | Butenyl-L-carnitine | 0.03 |  |  |
| C4:1-DC / C6 | Fumaryl-L-carnitine / Hexanoyl-L-carnitine | 0.08 | 0.2 | 6 |
| C5 | Valeryl-L-carnitine | 0.04 | 0.4 | 12 |
| C5-DC / C6-OH | Glutaryl-L-carnitine / Hydroxyhexanoyl-L-carnitine | 0.035 |  |  |
| C5-M-DC | Methylglutaryl-L-carnitine | 0.06 |  |  |
| C5:1 | Tiglyl-L-carnitine | 0.04 |  |  |
| C5:1-DC | Glutaconyl-L-carnitine | 0.015 |  |  |
| C6:1 | Hexenoyl-L-carnitine | 0.035 |  |  |
| C7-DC | Pimelyl-L-carnitine | 0.035 |  |  |
| C8 | Octanoyl-L-carnitine | 0.17 | 0.2 | 8 |
| C8:1 | Octenoyl-L-carnitine | 0.025 |  |  |
| C9 | Nonayl-L-carnitine | 0.04 |  |  |
| Amino acids |  |  |  |  |
| Arg | Arginine | 4 | 50 | 250 |
| Gln | Glutamine | 3 | 15 | 1000 |
| Gly | Glycine | 6 | 25 | 5000 |
| His | Histidine | 4 | 5 | 1000 |
| Met | Methionine | 1 | 10 | 400 |
| Orn | Ornithine | 5 | 10 | 1000 |
| Phe | Phenylalanine | 2 | 5 | 1000 |
| Pro | Proline | 2 | 10 | 1000 |
| Ser | Serine | 3 | 10 | 1000 |
| Thr | Threonine | 3 | 50 | 1000 |
| Trp | Tryptophan | 10 | 30 | 200 |
| Tyr | Tyrosine | 3 | 20 | 1000 |
| Val | Valine | 4 | 20 | 1000 |
| xLeu | Leucine/Isoleucin | 2 | 20 | 2000 |
| Glycerophospholipids |  |  |  |  |
| PC aa C24:0 | Phosphatidylcholine diacyl C 24:0 | 0.1 |  |  |
| PC aa C26:0 | Phosphatidylcholine diacyl C 26:0 | 1.4 |  |  |
| PC aa C28:1 | Phosphatidylcholine diacyl C 28:1 | 0.04 |  |  |
| PC aa C30:0 | Phosphatidylcholine diacyl C 30:0 | 0.2 |  |  |
| PC aa C30:2 | Phosphatidylcholine diacyl C 30:2 | 0.006 |  |  |
| PC aa C32:0 | Phosphatidylcholine diacyl C 32:0 | 0.04 |  |  |
| PC aa C32:1 | Phosphatidylcholine diacyl C 32:1 | 0.06 |  |  |
| PC aa C32:2 | Phosphatidylcholine diacyl C 32:2 | 0.03 |  |  |
| PC aa C32:3 | Phosphatidylcholine diacyl C 32:3 | 0.008 |  |  |
| PC aa C34:1 | Phosphatidylcholine diacyl C 34:1 | 0.06 |  |  |
| PC aa C34:2 | Phosphatidylcholine diacyl C 34:2 | 0.1 |  |  |
| PC aa C34:3 | Phosphatidylcholine diacyl C 34:3 | 0.01 |  |  |
| PC aa C34:4 | Phosphatidylcholine diacyl C 34:4 | 0.006 |  |  |
| PC aa C36:0 | Phosphatidylcholine diacyl C 36:0 | 0.2 |  |  |
| PC aa C36:1 | Phosphatidylcholine diacyl C 36:1 | 0.03 |  |  |
| PC aa C36:2 | Phosphatidylcholine diacyl C 36:2 | 0.15 |  |  |
| PC aa C36:3 | Phosphatidylcholine diacyl C 36:3 | 0.04 |  |  |
| PC aa C36:4 | Phosphatidylcholine diacyl C 36:4 | 0.04 |  |  |
| PC aa C36:5 | Phosphatidylcholine diacyl C 36:5 | 0.01 |  |  |
| PC aa C36:6 | Phosphatidylcholine diacyl C 36:6 | 0.015 |  |  |
| PC aa C38:0 | Phosphatidylcholine diacyl C 38:0 | 0.2 |  |  |
| PC aa C38:1 | Phosphatidylcholine diacyl C 38:1 | 0.08 |  |  |
| PC aa C38:3 | Phosphatidylcholine diacyl C 38:3 | 0.04 |  |  |
| PC aa C38:4 | Phosphatidylcholine diacyl C 38:4 | 0.03 |  |  |
| PC aa C38:5 | Phosphatidylcholine diacyl C 38:5 | 0.015 |  |  |
| PC aa C38:6 | Phosphatidylcholine diacyl C 38:6 | 0.02 |  |  |
| PC aa C40:1 | Phosphatidylcholine diacyl C 40:1 | 0.4 |  |  |
| PC aa C40:2 | Phosphatidylcholine diacyl C 40:2 | 0.02 |  |  |
| PC aa C40:3 | Phosphatidylcholine diacyl C 40:3 | 0.006 |  |  |
| PC aa C40:4 | Phosphatidylcholine diacyl C 40:4 | 0.01 |  |  |
| PC aa C40:5 | Phosphatidylcholine diacyl C 40:5 | 0.04 |  |  |
| PC aa C40:6 | Phosphatidylcholine diacyl C 40:6 | 1.2 |  |  |
| PC aa C42:0 | Phosphatidylcholine diacyl C 42:0 | 0.05 |  |  |
| PC aa C42:1 | Phosphatidylcholine diacyl C 42:1 | 0.008 |  |  |
| PC aa C42:2 | Phosphatidylcholine diacyl C 42:2 | 0.006 |  |  |
| PC aa C42:4 | Phosphatidylcholine diacyl C 42:4 | 0.006 |  |  |
| PC aa C42:5 | Phosphatidylcholine diacyl C 42:5 | 0.05 |  |  |
| PC aa C42:6 | Phosphatidylcholine diacyl C 42:6 | 0.3 |  |  |
| PC ae C30:0 | Phosphatidylcholine acyl-alkyl C 30:0 | 0.15 |  |  |
| PC ae C30:1 | Phosphatidylcholine acyl-alkyl C 30:1 | 0.02 |  |  |
| PC ae C30:2 | Phosphatidylcholine acyl-alkyl C 30:2 | 0.57 |  |  |
| PC ae C32:1 | Phosphatidylcholine acyl-alkyl C 32:1 | 0.009 |  |  |
| PC ae C32:2 | Phosphatidylcholine acyl-alkyl C 32:2 | 0.02 |  |  |
| PC ae C34:0 | Phosphatidylcholine acyl-alkyl C 34:0 | 0.017 |  |  |
| PC ae C34:1 | Phosphatidylcholine acyl-alkyl C 34:1 | 0.012 |  |  |
| PC ae C34:2 | Phosphatidylcholine acyl-alkyl C 34:2 | 0.01 |  |  |
| PC ae C34:3 | Phosphatidylcholine acyl-alkyl C 34:3 | 0.015 |  |  |
| PC ae C36:0 | Phosphatidylcholine acyl-alkyl C 36:0 | 0.12 |  |  |
| PC ae C36:1 | Phosphatidylcholine acyl-alkyl C 36:1 | 0.03 |  |  |
| PC ae C36:2 | Phosphatidylcholine acyl-alkyl C 36:2 | 0.01 |  |  |
| PC ae C36:3 | Phosphatidylcholine acyl-alkyl C 36:3 | 0.007 |  |  |
| PC ae C36:4 | Phosphatidylcholine acyl-alkyl C 36:4 | 0.013 |  |  |
| PC ae C36:5 | Phosphatidylcholine acyl-alkyl C 36:5 | 0.012 |  |  |
| PC ae C38:0 | Phosphatidylcholine acyl-alkyl C 38:0 | 0.066 |  |  |
| PC ae C38:1 | Phosphatidylcholine acyl-alkyl C 38:1 | 0.015 |  |  |
| PC ae C38:2 | Phosphatidylcholine acyl-alkyl C 38:2 | 0.018 |  |  |
| PC ae C38:3 | Phosphatidylcholine acyl-alkyl C 38:3 | 0.01 |  |  |
| PC ae C38:4 | Phosphatidylcholine acyl-alkyl C 38:4 | 0.015 |  |  |
| PC ae C38:5 | Phosphatidylcholine acyl-alkyl C 38:5 | 0.01 |  |  |
| PC ae C38:6 | Phosphatidylcholine acyl-alkyl C 38:6 | 0.03 |  |  |
| PC ae C40:0 | Phosphatidylcholine acyl-alkyl C 40:0 | 12 |  |  |
| PC ae C40:1 | Phosphatidylcholine acyl-alkyl C 40:1 | 0.06 |  |  |
| PC ae C40:2 | Phosphatidylcholine acyl-alkyl C 40:2 | 0.01 |  |  |
| PC ae C40:3 | Phosphatidylcholine acyl-alkyl C 40:3 | 0.015 |  |  |
| PC ae C40:4 | Phosphatidylcholine acyl-alkyl C 40:4 | 0.1 |  |  |
| PC ae C40:5 | Phosphatidylcholine acyl-alkyl C 40:5 | 0.006 |  |  |
| PC ae C40:6 | Phosphatidylcholine acyl-alkyl C 40:6 | 0.025 |  |  |
| PC ae C42:0 | Phosphatidylcholine acyl-alkyl C 42:0 | 0.4 |  |  |
| PC ae C42:1 | Phosphatidylcholine acyl-alkyl C 42:1 | 0.03 |  |  |
| PC ae C42:2 | Phosphatidylcholine acyl-alkyl C 42:2 | 0.006 |  |  |
| PC ae C42:3 | Phosphatidylcholine acyl-alkyl C 42:3 | 0.006 |  |  |
| PC ae C42:4 | Phosphatidylcholine acyl-alkyl C 42:4 | 0.3 |  |  |
| PC ae C42:5 | Phosphatidylcholine acyl-alkyl C 42:5 | 1.3 |  |  |
| PC ae C44:3 | Phosphatidylcholine acyl-alkyl C 44:3 | 0.006 |  |  |
| PC ae C44:4 | Phosphatidylcholine acyl-alkyl C 44:4 | 0.01 |  |  |
| PC ae C44:5 | Phosphatidylcholine acyl-alkyl C 44:5 | 0.02 |  |  |
| PC ae C44:6 | Phosphatidylcholine acyl-alkyl C 44:6 | 0.09 |  |  |
| lysoPC a C14:0 | lysoPhosphatidylcholine acyl C14:0 | 5 |  |  |
| lysoPC a C16:0 | lysoPhosphatidylcholine acyl C16:0 | 0.12 |  |  |
| lysoPC a C16:1 | lysoPhosphatidylcholine acyl C16:1 | 0.07 |  |  |
| lysoPC a C17:0 | lysoPhosphatidylcholine acyl C17:0 | 0.05 |  |  |
| lysoPC a C18:0 | lysoPhosphatidylcholine acyl C18:0 | 0.05 |  |  |
| lysoPC a C18:1 | lysoPhosphatidylcholine acyl C18:1 | 0.1 |  |  |
| lysoPC a C18:2 | lysoPhosphatidylcholine acyl C18:2 | 0.1 |  |  |
| lysoPC a C20:3 | lysoPhosphatidylcholine acyl C20:3 | 0.2 |  |  |
| lysoPC a C20:4 | lysoPhosphatidylcholine acyl C20:4 | 0.02 |  |  |
| lysoPC a C24:0 | lysoPhosphatidylcholine acyl C24:0 | 1.3 |  |  |
| lysoPC a C26:0 | lysoPhosphatidylcholine acyl C26:0 | 0.5 |  |  |
| lysoPC a C26:1 | lysoPhosphatidylcholine acyl C26:1 | 4 |  |  |
| lysoPC a C28:0 | lysoPhosphatidylcholine acyl C28:0 | 0.3 |  |  |
| lysoPC a C28:1 | lysoPhosphatidylcholine acyl C28:1 | 0.15 |  |  |
| lysoPC a C6:0 | lysoPhosphatidylcholine acyl C6:0 | 0.04 |  |  |
| Sphingolipids |  |  |  |  |
| SM (OH) C14:1 | Hydroxysphingomyelin C 14:1 | 0.025 |  |  |
| SM (OH) C16:1 | Hydroxysphingomyelin C 16:1 | 0.012 |  |  |
| SM (OH) C22:1 | Hydroxysphingomyelin C 22:1 | 0.015 |  |  |
| SM (OH) C22:2 | Hydroxysphingomyelin C 22:2 | 0.01 |  |  |
| SM (OH) C24:1 | Hydroxysphingomyelin C 24:1 | 0.01 |  |  |
| SM C16:0 | Sphingomyelin C 16:0 | 0.03 |  |  |
| SM C16:1 | Sphingomyelin C 16:1 | 0.01 |  |  |
| SM C18:0 | Sphingomyelin C 18:0 | 0.07 |  |  |
| SM C18:1 | Sphingomyelin C 18:1 | 0.01 |  |  |
| SM C20:2 | Sphingomyelin C 20:2 | 0.005 |  |  |
| SM C22:3 | Sphingomyelin C 22:3 | 0.01 |  |  |
| SM C24:0 | Sphingomyelin C 24:0 | 0.13 |  |  |
| SM C24:1 | Sphingomyelin C 24:1 | 0.035 |  |  |
| SM C26:0 | Sphingomyelin C 26:0 | 0.015 |  |  |
| SM C26:1 | Sphingomyelin C 26:1 | 0.006 |  |  |
| Sugars |  |  |  |  |
| H1 | Hexose | 20 | 200 | 2000 |

Abbreviations: LOD, limit of detection; LLOQ, lower limit of quantification; ULOQ, upper limit of quantification.

^a^The quantification range was determined by BIOCRATES and adopted from the Manual: “Absolute*IDQ*^TM^ p150 kit – Analytical Specifications” (BIOCRATES Life Sciences AG, Innsbruck, Austria).
