# Supplementary material for: Reliability of Serum Metabolite Concentrations over a 4-Month Period Using a Targeted Metabolomic Approach
Source: PLoS One. 2011 Jun 15;6(6):e21103. doi: 10.1371/journal.pone.0021103 (PMC3115978; doi:10.1371/journal.pone.0021103)
Supplement: Table S2 — Analytical Variance of 163 Metabolites Measured with the BIOCRATES Absolute IDQ Targeted Metabolomics Technology. Footnote: Abbreviations: CV, coefficient of variation; LOD, limit of detection. aAnalytical variance was determined by measuring 5 replicates on each of the 46 plates containing the EPIC-samples (total of 230 replicates) and reported as CV%. Note: higher CVs are mainly observed for metabolites that show very low concentrations and are below the LOD of the assay. bMetabolite concentration was below the assay's LOD. (DOCX) [file pone.0021103.s002.docx]

| Table S2: Analytical variance of 163 metabolites measured with the BIOCRATES Absolute IDQ targeted metabolomics technology | | | |
| --- | --- | --- | --- |
| ***Metabolite*** | ***Within-plate CV (%)^a^*** | ***Between-plate CV (%)^a^ ntification Range*** |  |
| Acylcarnitines |  |  |  |
| C0 | 5.1 | 1.6 |  |
| C10 | 5.6 | 11.4 |  |
| C10:1^b^ | 8.9 | 11.7 |  |
| C10:2 | 11.4 | 30.2 |  |
| C12^b^ | 6.8 | 10.4 |  |
| C12-DC^b^ | 10.2 | 13.0 |  |
| C12:1^b^ | 11.9 | 7.7 |  |
| C14^b^ | 8.5 | 15.3 |  |
| C14:1 | 7.2 | 18.7 |  |
| C14:1-OH^b^ | 14.1 | 30.2 |  |
| C14:2 | 11.2 | 17.4 |  |
| C14:2-OH | 18.4 | 50.5 |  |
| C16 | 7.7 | 9.0 |  |
| C16-OH^b^ | 26.3 | 55.0 |  |
| C16:1^b^ | 9.8 | 17.5 |  |
| C16:1-OH^b^ | 18.5 | 39.7 |  |
| C16:2 | 22.3 | 48.0 |  |
| C16:2-OH^b^ | 18.7 | 44.4 |  |
| C18 | 13.8 | 12.8 |  |
| C18:1 | 7.1 | 3.9 |  |
| C18:1-OH^b^ | 23.2 | 46.9 |  |
| C18:2 | 10.0 | 10.2 |  |
| C2 | 4.5 | 4.0 |  |
| C3 | 7.4 | 12.5 |  |
| C3-DC / C4-OH^b^ | 20.2 | 31.3 |  |
| C3-DC-M / C5-OH | 17.1 | 47.3 |  |
| C3-OH^b^ | 15.1 | 37.4 |  |
| C3:1^b^ | 20.7 | 86.1 |  |
| C4 | 29.3 | 94.8 |  |
| C4:1^b^ | 8.8 | 25.2 |  |
| C4:1-DC / C6 | 16.6 | 83.3 |  |
| C5 | 23.3 | 95.9 |  |
| C5-DC / C6-OH | 8.4 | 17.6 |  |
| C5-M-DC^b^ | 10.2 | 60.4 |  |
| C5:1^b^ | 14.9 | 36.7 |  |
| C5:1-DC^b^ | 15.8 | 49.1 |  |
| C6:1^b^ | 17.0 | 76.4 |  |
| C7-DC | 10.7 | 26.1 |  |
| C8^b^ | 6.2 | 12.4 |  |
| C8:1 | 8.5 | 22.3 |  |
| C9 | 8.5 | 24.4 |  |
| Amino acids |  |  |  |
| Arg | 6.1 | 3.4 |  |
| Gln | 6.7 | 6.3 |  |
| Gly | 6.7 | 4.4 |  |
| His | 7.0 | 6.7 |  |
| Met | 7.2 | 6.5 |  |
| Orn | 8.7 | 4.8 |  |
| Phe | 6.7 | 6.2 |  |
| Pro | 5.3 | 3.7 |  |
| Ser | 8.0 | 15.3 |  |
| Thr | 8.2 | 16.6 |  |
| Trp | 5.8 | 6.6 |  |
| Tyr | 8.3 | 5.4 |  |
| Val | 7.8 | 4.3 |  |
| xLeu | 7.5 | 4.5 |  |
| Glycerophospholipids |  |  |  |
| PC aa C24:0 | 35.9 | 65.2 |  |
| PC aa C26:0^b^ | 27.9 | 56.2 |  |
| PC aa C28:1 | 5.4 | 8.2 |  |
| PC aa C30:0 | 5.1 | 4.3 |  |
| PC aa C30:2 | 69.9 | 88.6 |  |
| PC aa C32:0 | 5.7 | 6.2 |  |
| PC aa C32:1 | 6.5 | 13.7 |  |
| PC aa C32:2 | 7.2 | 6.4 |  |
| PC aa C32:3 | 7.4 | 6.5 |  |
| PC aa C34:1 | 5.4 | 9.5 |  |
| PC aa C34:2 | 6.4 | 15.9 |  |
| PC aa C34:3 | 5.5 | 9.1 |  |
| PC aa C34:4 | 6.8 | 6.9 |  |
| PC aa C36:0 | 8.5 | 15.9 |  |
| PC aa C36:1 | 5.2 | 2.6 |  |
| PC aa C36:2 | 5.4 | 11.2 |  |
| PC aa C36:3 | 5.0 | 7.7 |  |
| PC aa C36:4 | 5.9 | 7.9 |  |
| PC aa C36:5 | 6.5 | 6.1 |  |
| PC aa C36:6 | 6.9 | 6.2 |  |
| PC aa C38:0 | 6.5 | 11.4 |  |
| PC aa C38:1 | 27.2 | 25.1 |  |
| PC aa C38:3 | 5.2 | 3.0 |  |
| PC aa C38:4 | 5.1 | 4.8 |  |
| PC aa C38:5 | 5.5 | 6.3 |  |
| PC aa C38:6 | 4.9 | 5.2 |  |
| PC aa C40:1^b^ | 10.3 | 6.0 |  |
| PC aa C40:2 | 12.8 | 13.4 |  |
| PC aa C40:3 | 8.4 | 9.5 |  |
| PC aa C40:4 | 5.5 | 3.5 |  |
| PC aa C40:5 | 4.8 | 3.5 |  |
| PC aa C40:6 | 4.9 | 3.5 |  |
| PC aa C42:0 | 6.9 | 3.7 |  |
| PC aa C42:1 | 9.2 | 7.8 |  |
| PC aa C42:2 | 10.7 | 10.5 |  |
| PC aa C42:4 | 11.5 | 8.2 |  |
| PC aa C42:5 | 8.2 | 3.9 |  |
| PC aa C42:6 | 6.5 | 6.3 |  |
| PC ae C30:0 | 9.2 | 11.6 |  |
| PC ae C30:1 | 23.8 | 42.4 |  |
| PC ae C30:2 | 18.1 | 24.8 |  |
| PC ae C32:1 | 7.4 | 6.1 |  |
| PC ae C32:2 | 6.8 | 7.5 |  |
| PC ae C34:0 | 7.1 | 5.5 |  |
| PC ae C34:1 | 5.4 | 6.6 |  |
| PC ae C34:2 | 5.4 | 7.6 |  |
| PC ae C34:3 | 5.5 | 7.0 |  |
| PC ae C36:0 | 13.6 | 19.0 |  |
| PC ae C36:1 | 5.9 | 7.3 |  |
| PC ae C36:2 | 6.3 | 6.0 |  |
| PC ae C36:3 | 7.0 | 6.2 |  |
| PC ae C36:4 | 5.6 | 7.7 |  |
| PC ae C36:5 | 5.7 | 8.4 |  |
| PC ae C38:0 | 6.1 | 11.4 |  |
| PC ae C38:1 | 14.8 | 17.6 |  |
| PC ae C38:2 | 9.3 | 7.1 |  |
| PC ae C38:3 | 5.7 | 5.4 |  |
| PC ae C38:4 | 5.2 | 6.5 |  |
| PC ae C38:5 | 5.2 | 5.8 |  |
| PC ae C38:6 | 5.5 | 5.2 |  |
| PC ae C40:0^b^ | 3.8 | 11.5 |  |
| PC ae C40:1 | 6.8 | 12.4 |  |
| PC ae C40:2 | 6.4 | 4.6 |  |
| PC ae C40:3 | 8.9 | 4.2 |  |
| PC ae C40:4 | 5.8 | 9.3 |  |
| PC ae C40:5 | 5.4 | 4.0 |  |
| PC ae C40:6 | 5.1 | 4.9 |  |
| PC ae C42:0^b^ | 9.1 | 14.1 |  |
| PC ae C42:1 | 13.7 | 15.3 |  |
| PC ae C42:2 | 7.6 | 5.8 |  |
| PC ae C42:3 | 8.5 | 6.7 |  |
| PC ae C42:4 | 7.1 | 2.8 |  |
| PC ae C42:5 | 5.3 | 1.3 |  |
| PC ae C44:3 | 21.1 | 12.5 |  |
| PC ae C44:4 | 9.1 | 4.6 |  |
| PC ae C44:5 | 6.0 | 2.6 |  |
| PC ae C44:6 | 5.9 | 1.3 |  |
| lysoPC a C14:0 | 4.0 | 12.0 |  |
| lysoPC a C16:0 | 5.0 | 12.6 |  |
| lysoPC a C16:1 | 6.6 | 11.7 |  |
| lysoPC a C17:0 | 7.3 | 13.9 |  |
| lysoPC a C18:0 | 5.3 | 13.7 |  |
| lysoPC a C18:1 | 5.3 | 11.1 |  |
| lysoPC a C18:2 | 5.4 | 8.4 |  |
| lysoPC a C20:3 | 7.9 | 9.5 |  |
| lysoPC a C20:4 | 7.4 | 10.0 |  |
| lysoPC a C24:0^b^ | 26.6 | 43.7 |  |
| lysoPC a C26:0^b^ | 39.1 | 65.9 |  |
| lysoPC a C26:1^b^ | 11.4 | 14.8 |  |
| lysoPC a C28:0^b^ | 28.1 | 45.6 |  |
| lysoPC a C28:1 | 20.5 | 34.7 |  |
| lysoPC a C6:0^b^ | 52.3 | 68.3 |  |
| Sphingolipids |  |  |  |
| SM (OH) C14:1 | 6.8 | 14.3 |  |
| SM (OH) C16:1 | 6.8 | 17.4 |  |
| SM (OH) C22:1 | 6.8 | 21.9 |  |
| SM (OH) C22:2 | 7.0 | 19.2 |  |
| SM (OH) C24:1 | 8.3 | 22.6 |  |
| SM C16:0 | 6.3 | 16.4 |  |
| SM C16:1 | 8.9 | 14.3 |  |
| SM C18:0 | 7.2 | 16.7 |  |
| SM C18:1 | 6.5 | 15.9 |  |
| SM C20:2 | 21.0 | 23.0 |  |
| SM C22:3 | 89.8 | 53.8 |  |
| SM C24:0 | 6.8 | 22.2 |  |
| SM C24:1 | 7.2 | 23.0 |  |
| SM C26:0 | 20.1 | 25.9 |  |
| SM C26:1 | 11.4 | 20.9 |  |
| Sugars |  |  |  |
| H1 | 4.1 | 2.0 |  |
| Median | 7.3 | 11.4 |  |

Abbreviations: CV, coefficient of variation; LOD, limit of detection.

^a^Analytical variance was determined by measuring 5 replicates on each of the 46 plates containing the EPIC-samples (total of 230 replicates) and reported as CV%. Note: higher CVs are mainly observed for metabolites that show very low concentrations and are below the LOD of the assay.

^b^Metabolite concentration was below the assay’s LOD.
